# People and water: Exploring the social-ecological condition of watersheds of the United States

**DOI:** 10.1525/elementa.189

**Published:** 2017

**Authors:** Murray W. Scown, Joseph E. Flotemersch, Trisha L. Spanbauer, Tarsha Eason, Ahjond Garmestani, Brian C. Chaffin

**Affiliations:** * Lund University Centre for Sustainability Studies, Lund, 22362, SE; † National Exposure Research Laboratory, US; ‡ Department of Integrative Biology, University of Texas at Austin, Austin, TX 78712, US; § National Risk Management Research Laboratory, US; ‖ W.A. Franke College of Forestry and Conservation, University of Montana, Missoula, MT 59812, US

**Keywords:** ecosystem services, spatial patterns, social-ecological systems, sustainability, governance

## Abstract

A recent paradigm shift from purely biophysical towards social-ecological assessment of watersheds has been proposed to understand, monitor, and manipulate the myriad interactions between human well-being and the ecosystem services that watersheds provide. However, large-scale, quantitative studies in this endeavour remain limited. We utilised two newly developed ‘big-data’ sets—the Index of Watershed Integrity (IWI) and the Human Well-Being Index (HWBI)—to explore the social-ecological condition of watersheds throughout the conterminous U.S., and identified environmental and socio-economic influences on watershed integrity and human well-being. Mean county IWI was highly associated with ecoregion, industry-dependence, and state, in a spatially-explicit regression model (R^2^ = 0.77, *P* < 0.001), whereas HWBI was not (R^2^ = 0.31, *P* < 0.001). HWBI is likely influenced by factors not explored here, such as governance structure and formal and informal organisations and institutions. ‘Win-win’ situations in which both IWI and HWBI were above the 75^th^ percentile were observed in much of Utah, Colorado, and New Hampshire, and lessons from governance that has resulted in desirable outcomes might be learnt from here. Eastern Kentucky and West Virginia, along with large parts of the desert southwest, had intact watersheds but low HWBI, representing areas worthy of further investigation of how ecosystem services might be utilised to improve well-being. The Temperate Prairies and Central USA Plains had widespread areas of low IWI but high HWBI, likely a result of historic exploitation of watershed resources to improve well-being, particularly in farming-dependent counties. The lower Mississippi Valley had low IWI and HWBI, which is likely related to historical (temporal) and upstream (spatial) impacts on both watershed integrity and well-being. The results emphasise the importance of considering spatial and temporal trade-offs when utilising the ecosystem services provided by watersheds to improve human well-being.

## Introduction

People are inextricably connected to freshwater resources, and the control of these resources has performed a unique role in the development of societies and economies worldwide (Postel and Richter, 2012). The coevolution of societies and freshwater environments has resulted in complex social-ecological systems in which humans have shaped the physical form of rivers (Ashmore, 2015), the spatiotemporal distribution of freshwater resources (Vörösmarty et al., 2010), and the structure and function of the ecosystems that occur in these environments (Ormerod et al., 2010). Humans have also become dependent upon the services that freshwater environments provide to society and must manage systems in a way that assures the sustainable provisioning of these services (Falkenmark, 2003). Central to understanding watersheds and effectively managing these complex social-ecological systems is the idea that humans are part of the system and not external to it (Callicott et al., 1999).

Inherent in all social-ecological systems are opportunities and trade-offs. Watersheds provide a range of resources and services that benefit humans and, in using these services to maintain or increase their well-being, humans may change the structure and function of a watershed (Parsons et al., 2016). Trade-offs occur when an increase in one ecosystem service provided by watersheds, for example crop production, results in a decline in another, such as water quality (Qiu and Turner, 2013). Similarly, utilisation or exploitation of one service by humans can limit the provisioning of other services in space and time (Tallis et al., 2008). Although humans frequently benefit from the services provided by watersheds (Qiu and Turner, 2013), simply because a watershed could provide a certain service does not imply that it is being utilised to increase human well-being, and such instances may present opportunities for socio-economic development. In addition, exploitation of watershed resources and services may not provide local benefits, but rather distant benefits at the cost of local degradation. Thus, a distinction exists between people living in a watershed versus people living outside that watershed, as well as between the beneficiaries of watershed exploitation and those who are disadvantaged by it. These actors and interactions result in complexity in the costs and benefits of watershed exploitation in space and time (e.g., Dalin et al., 2014; Liu et al., 2016).

Recognition of the complex interconnectedness of people and watersheds, including benefits, disadvantages, trade-offs, and opportunities, has engendered a paradigm shift for river science away from purely biophysical or ecological research towards coupled social-ecological assessment of these systems (Parsons et al., 2016). However, applications of such assessments remain in their infancy. Research to date has largely been theoretical, qualitative or limited to small scales (e.g., Nemec et al., 2014; Parsons et al., 2016; Rathwell and Peterson, 2012) with some exceptions from the public sector (Stenekes et al., 2010). Thus, quantitative, large-scale application of the new social-ecological paradigm for river science has been limited. Without quantitative assessment of the social-ecological condition of watersheds, pressing goals to quantify and understand trade-offs and interactions among ecosystem services and human well-being (Carpenter et al., 2009; Qiu and Turner, 2013) are unattainable in these complex systems.

‘Big data’—voluminous, varied, and spatiotemporally extensive data (sensu Miller and Goodchild, 2015)—may help to overcome some current limitations to the social-ecological assessment of watersheds. In the United States and other countries big data on the condition of watersheds and communities are becoming increasingly available (e.g., Summers et al., 2014; Thornbrugh et al., under review). Although these big data are in their initial stages, they present an opportunity to examine the physical condition of watersheds alongside the well-being of the people who live there, which has not been quantitatively attempted at large scales before.

The lack of large-scale, quantitative, social-ecological analyses of watersheds means that we do not know where or how stakeholders may, or should, begin to seek opportunities to utilise watershed resources in the hope of improving local well-being. Nor do we have a good understanding of how social and watershed conditions interact and influence future trajectories, which is essential for implementing effective watershed management in these areas. In this study, we explore the social-ecological condition of watersheds of the conterminous United States using newly developed indices of watershed integrity and human well-being. We hypothesise that areas with good social and watershed conditions exist, as do areas with poor conditions in both, and that these areas are not random but determined by spatial (geographical) and temporal (historical) factors. We attempt to interpret possible determinants of social-ecological watershed conditions by examining spatially-explicit associations between the two indices and other environmental and socio-economic factors including ecoregion, county industry dependence, and state. We also anticipate areas where historical practises have diminished watershed integrity to the benefit of economic development and human well-being, as well as areas with relatively intact watersheds but poor social conditions. We use the analyses to discuss the sustainable and unsustainable use of watershed services in certain parts of the United States and the potential to protect, promote, and utilise services from intact watersheds for the benefit of human well-being. We also discuss areas where the costs of watershed exploitation are borne by communities distant in space and time from those who benefit. Our findings highlight the importance of identifying the characteristics of these areas as a first step in targeting detailed research and intervention into social-ecological watershed processes and management.

## The indices

The Index of Watershed Integrity (IWI; Flotemersch et al., 2015; Thornbrugh et al., under review) was used in this study as an indicator of the level of anthropogenic alteration to surface watersheds throughout the conterminous U.S. The IWI is an aggregate index ranging from zero (low integrity) to one (high integrity) based on risk factors of the impairment of six key functions of watersheds; hydrologic regulation, regulation of water chemistry, sediment regulation, hydrologic connectivity, temperature regulation, and habitat provision (please see Flotemersch et al., 2015 for details). The IWI is a cumulative index in which all conditions upstream influence the score of a downstream reach. That is, impairment of a watershed can accumulate (or be cumulatively buffered) downstream, reflecting the reality of watershed-scale processes. The Index of Catchment Integrity (ICI; Thornbrugh et al., under review), which indicates local impairment independently of upstream impairment, is also available; however, we chose the IWI because it provides a more realistic perspective of upstream-downstream relationships in watersheds for the purpose of this study. In addition, we found that the mean county IWI was highly correlated with mean county ICI for the conterminous U.S. (Spearman's rho = 0.98, *P* < 0.001). The spatial unit of resolution for the IWI is the ‘catchment’ level of the National Hydrography Dataset (NHDPlus Version 2; McKay et al., 2012), of which approximately 2.6 million exist throughout the conterminous U.S. The IWI and ICI are not currently available for Alaska and Hawaii. We used the most up-to-date version of the IWI as of July 14, 2017; however, it is likely that continual updates will be made to new versions of the IWI as improvements to the calculation method and input data are made.

The Human Well-Being Index (HWBI; Summers et al., 2014) was used in this study as an indicator of people's well-being within communities throughout the U.S. The HWBI is an aggregate index ranging from zero (low well-being) to 100 (high well-being) based on the presence or provisioning of social, economic, and ecosystem services that benefit people living within an area. These services include activism, communication, community initiatives, education services, emergency preparedness, family services, healthcare, justice, labor, public works, capital investment, consumption, employment, finance, innovation, production, redistribution, air quality, food and fibre provisioning, green space, water quality, and water quantity (please see Summers et al., 2014 for details). The HWBI was chosen instead of other indices, such as the United Nations' Human Development Index (HDI) or Gross Domestic Product (GDP), because it does not focus purely on economic or socio-economic components but incorporates the three pillars of sustainability—environmental, economic, and social—in its evaluation (Summers et al., 2014). The spatial unit of resolution for the HWBI is the county level and the 2000–2010 decadal index was used in this study.

In order to capture potential environmental and socio-economic influences on watershed integrity and human well-being, three additional variables were included in the analysis; namely ecoregion, industry dependence, and state. Ecoregion was represented by the North American Level II Ecoregions (USEPA, 2012), which broadly characterise environmental conditions throughout North America. Industry dependence—the dependence of a county on a particular industry—was captured through use of the 2015 U.S. Department of Agriculture Economic Research Service County Typology Codes (USDA, 2015), which identify particular industries upon which counties are economically dependent. We incorporated state because of the potential importance of economic and legislative differences among states. We also used the Shannon Diversity Index (Shannon, 1948) to indicate the diversity of county industry-dependence within each state. Whilst we acknowledge the importance of good governance and strong formal institutions in enhancing both environmental condition (i.e., watershed integrity in this case) and human well-being, this early stage of large-scale social-ecological watershed research was limited to potential environmental (ecoregions), industrial (county industry typology), and governmental (state) drivers. In addition, we focused on whether or not a relationship existed and did not attempt to numerically quantify precisely what it was about these external factors that may be affecting IWI or HWBI.

**Figure 1:**
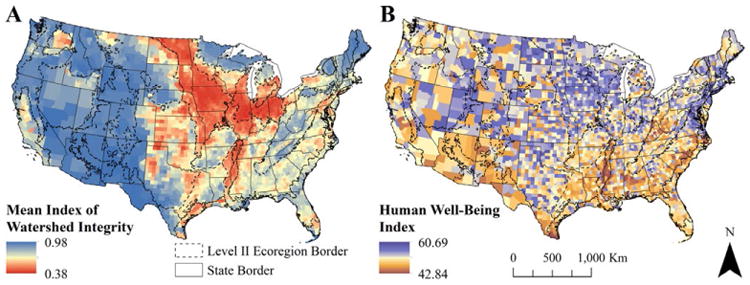
County-level maps of watershed integrity and human well-being **(A)** Mean Index of Watershed Integrity within each county and **(B)** The Human Well-Being Index for each county of the conterminous United States. North American Level II Ecoregion borders are also displayed. DOI: https://doi.org/10.1525/elementa.189.f1

This study was conducted on the 3108 counties throughout the conterminous U.S. (Figure 1). Agreement between the different spatial units was achieved by aggregating catchment IWI scores to the county level. A mean IWI was calculated for each county by spatially-weighted averaging of catchment IWI scores that had previously been generalised to a 250 m × 250 m grid for the entire conterminous U.S. That is, the IWI score was determined for every 250 m × 250 m square of land in the conterminous U.S. from the catchment polygon within which that land fell and stored in raster format. Then, the average IWI of all 250 m × 250 m squares within each county was calculated. While a minor loss of precision occurs from generalising IWI to a 250 m × 250 m grid, this approach was taken to reduce computer processing time. All counties that fell entirely within an ecoregion were attributed to that ecoregion, while counties crossing the boundary of multiple ecoregions were attributed to the ecoregion that occupied the largest areas within that county. All other variables were already resolved to the county level. Future local-scale studies of this type would benefit from maintaining the high spatial precision of catchment and ecoregion information, as well as including within-county social information, such as urban-rural typologies, which were beyond the scope of this study due to computing limitations.

We use the IWI and HWBI at the county level acknowledging several caveats. First, both are aggregated indices composed of many, but not all, factors influencing their respective topics. Thus, interpreting what drives higher or lower values of each index can be difficult, and other important factors, such as inequality, can be overlooked. Factors, or sub-indices, are unweighted in these indices, meaning that all are considered equally important in determining the overall condition. This is appropriate for a general indication of system conditions; however, can be misleading when considering a particular system attribute or part. For example, the presence of dams can be highly detrimental to the hydrology of streams and rivers; however, if all other aspects of the IWI, such as land use, soils, wastewater treatment, are “good”, the overall watershed index will remain relatively high. Second, in aggregating the IWI to the county level, all variability within counties is lost. Large counties may have highly variable IWI because many watersheds can occur within county boundaries. Thus, while one watershed in a particular county may be heavily impaired, the mean IWI for that county may still remain high if others are intact. This is a problem inevitably faced when dealing with data that are resolved to different spatial units, which limits the transferability of large-scale results to local scales. Additionally, population density is highly variable between urban and rural areas within counties, and watershed impairment in rural areas may actually provide benefits to well-being in urban areas. Within-county analysis was beyond the scope of this study but is important future research. Third, high HWBI values may be indicative of good governance and the provision of social services, which are important for well-being in a general sense, but may not account for or capture the actual well-being felt by individuals in any particular county, which is much harder to measure at large scales. This raises questions about the role of formal institutions, including the government, church, and private sectors, in determining individuals' well-being. Additionally, no distinction is made among different socio-economic groups within a county, such as elites, disadvantaged, or indigenous persons, whose situations may not be well-represented by the U.S.-wide HWBI (Smith et al., 2015). While we acknowledge these caveats in the IWI and HWBI, we believe their advantages of being 1) multidimensional, 2) intended to inform policy-makers, and 3) spatially-explicit and available over the conterminous U.S., outweigh the limitations in interpreting them and present a new opportunity to begin to quantitatively explore watersheds as social-ecological systems over large scales.

## Statistical methods

Spatial structure, relationships, and autocorrelation are ubiquitous in geographic data, such as those used in this study, which presents a nuisance for traditional statistical approaches but also an opportunity to quantify and incorporate spatial structure into subsequent analyses (Legendre, 1993). Spatial autocorrelation was present in both the mean IWI and HWBI among counties of the conterminous U.S. (both Moran's *I* > 0.34, *P* < 0.001). We used Moran's Eigenvector Maps (MEMs; Dray et al., 2006) to quantify the spatial structure present in the data. MEMs are an extension of the principal coordinate analyses of neighbour matrices (PCNM) proposed by (Borcard and Legendre 2002), which decompose a matrix of neighbouring sample units (in this study, counties) into a set of orthogonal eigenvectors representing all scales of spatial structure in the data. However, where PCNM maximises the eigenvalue associated with each eigenvector, MEMs maximise Moran's index of spatial autocorrelation (*I*) in eigenvector elements among the sample units. The large number of MEMs was reduced by regressing each MEM onto each index (IWI and HWBI) and plotting the coefficients of determination in descending order for each index. The maximum number of MEMs to include in further IWI and HWBI analyses was taken to be the first inflection point on these plots (Figure S1A and B). This inflection point indicates where no substantial additional variance in the indices would be captured by including additional MEMs.

The relationships between the two indices and the environmental and socio-economic variables were then determined using spatial multiple linear regression. Regression models took the form of:

(1) $$y=X\beta_{X}+Z\beta_{Z}+ɛ$$

where *y* is a vector of either IWI or HWBI for all counties, X is a matrix of independent environmental and socio-economic variables with associated parameter estimates *β*_x_, Z is a matrix of the reduced set of MEMs (variables quantifying the spatial structure among counties) with associated parameter estimates *β*_z_, and *ε* is the residual error. The inclusion of spatial terms in a regression analysis of spatially-autocorrelated data is essential to avoid violating the assumption of independence, to improve the standard errors or parameter estimates, and to reduce Type I and Type II errors. Finally, the variance in each of the two indices explained by the independent variables alone, the spatial structure alone, and the independent variables and spatial structure in combination was ‘partialled’ out using the approach described by Legendre (1993).

## Environmental and socio-economic influences on watershed integrity and human well-being

**Figure 2:**
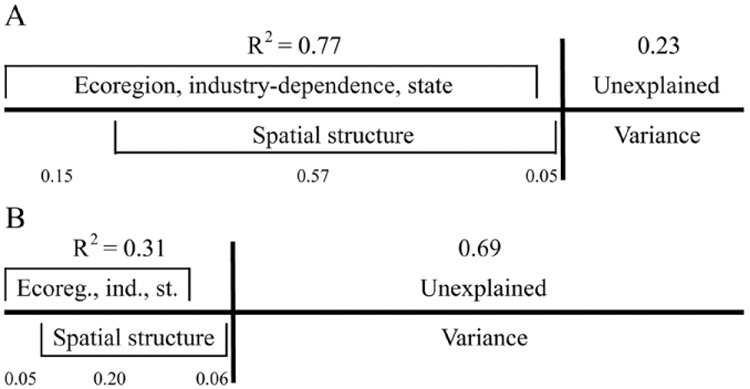
Partitioning of covariate and spatial influences on watershed integrity and human well-being Decomposition of variance in **(A)** Mean IWI and **(B)** HWBI explained by environmental and socio-economic variables (North American Level II Ecoregion, USDA county industry-dependence, state), and spatial structure in the spatial regression models. DOI: https://doi.org/10.1525/elementa.189.f2

A total of 77% of the variance in the mean county IWI could be explained by ecoregion, industry-dependence, state, and a reduced set of spatial variables from the MEMs, which quantify the spatial autocorrelation in the index, throughout the conterminous U.S. (Figure 2A). Almost all of the variance explained by spatial structure among counties coincided with that of the independent variables, indicating that ecoregion, industry-dependence, and state capture much of the spatial structure present in the IWI. Much less variance in the county HWBI was explained by ecoregion, industry-dependence, state, and the best reduced set of spatial variables from the MEMs, with R^2^ = 0.31 (Figure 2B).

When partitioning the nation into ecoregions, nine of the twenty ecoregions had a significant negative effect on mean county IWI, with the Central USA Plains and Temperate Prairies having the strongest effect (Figure S2A). These two ecoregions are heavily modified by agriculture, which reduces watershed integrity through clearing of natural vegetation, channelisation of watercourses, and various other mechanisms (Allan et al., 1997; Schilling et al., 2008). The environmental conditions in these ecoregions are particularly amenable to large-scale, intensive agriculture, resulting in an indirect effect of ecoregion on mean IWI through the effects of farming in these areas. Lower mean IWI in farming-dependent counties (Figure S2B) reinforced this finding.

With regard to industry-dependence, counties dependent on the recreation industry had the highest IWI, on average (Figure S2B), reflecting the importance of watershed intactness for various recreational pursuits. State appeared to have less of an effect on mean county IWI than either ecoregion or industry-dependence, although Maine, New Mexico, and Florida each had a significant positive effect on mean IWI, based on the spatial regression model (Figure S2C). However, mean county IWI was extremely variable within most states (Figure S2C).

Effects of ecoregion, industry-dependence, and state on the HWBI were much less evident. HWBI varied far more widely within ecoregions and within states, than median HWBI varies between ecoregions or between states. All ecoregions had a similar median HWBI, although the range in HWBI within ecoregions varied greatly (Figure S3A), largely in relation to ecoregion size. Government-dependent counties had lower HWBI than others, on average (Figure S3B). Variance in the HWBI within most states was high (Figure S3C), which suggests that factors operating at a smaller scale than state are influencing well-being. Overall, Utah had the highest median HWBI among counties while South Carolina had the lowest.

Correlations between mean county IWI and the HWBI and its service subindices were weak, with all absolute Spearman's rank correlation coefficients (|rho|) less than 0.2 (Figure S4). Rank correlation between mean IWI and the HWBI service subindices was greatest for green space (rho = 0.19, *P* < 0.001), followed by water quality (rho = 0.16, *P* < 0.001). Although weak, these correlations indicate a general increase in these two ecosystem services with increasing watershed integrity. Overall, the correlation between mean IWI and the HWBI among counties of the conterminous U.S. was negative (rho = −0.17, *P* < 0.001), which suggests there is a weak general decline in well-being with increasing watershed integrity. This likely reflects that many watersheds have historically been impaired by various mechanisms in order to increase well-being; however, conclusions are not possible from our results about whether well-being has increased in the same area where watersheds have been exploited, or in distant or urban areas. Concomitantly, many watersheds with high integrity are protected (e.g., large tracts of public land in the U.S. west), which largely prevents the industrial exploitation of watershed-supported ecosystem services in order to increase human well-being at large scales (e.g., watershed development for industry or agriculture).

## Spatial patterns in social-ecological watershed condition

**Figure 3:**
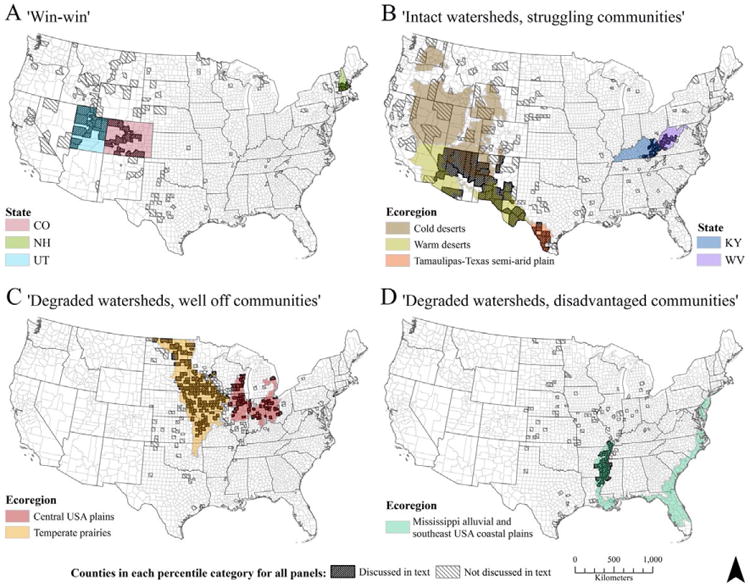
Hotspots and coldspots of watershed integrity and human well-being Counties of the conterminous U.S. that fall in each of the four outlying percentile categories. **(A)** Mean IWI and HWBI above the 75^th^ percentile; **(B)** Mean IWI above the 75^th^ percentile and HWBI below the 25^th^ percentile; **(C)** Mean IWI below the 25^th^ percentile and HWBI above the 75^th^ percentile; and **(D)** Mean IWI and HWBI below the 25^th^ percentile. Clusters of counties with non-random patterns associated with either ecoregion, industry-dependence, or state are darkly striped and discussed in the text. Counties that fell within each category but were not distinctly clustered in association with the independent variables examined here are lightly striped and not discussed further. Particular ecoregions and states with which clusters of darkly striped counties were associated are highlighted in each panel. DOI: https://doi. org/10.1525/elementa.189.f3

Spatial patterns in the social-ecological condition of watersheds were examined by identifying those counties that fell either below the 25^th^ percentile or above the 75^th^ percentile of both the mean IWI and the HWBI of all 3108 counties throughout the conterminous U.S. Examination of upper and lower percentiles is an effective way of identifying “hotspots” and “coldspots” of particular interest in space (Qiu and Turner, 2013). If the spatial distribution of counties in these four categories (win-win; intact watersheds, struggling communities; degraded watersheds, well-off communities; degraded watersheds, disadvantaged communities) was random, approximately 6% of counties in each ecoregion, industry-dependence class, and state would be expected to occur in each of the categories. However, proportions as much as ten times the expected were observed in some areas (Figure 3), indicating that spatially-structured independent drivers are influencing the social-ecological condition of watersheds throughout the conterminous U.S.

‘Win-win’ situations (high mean IWI and HWBI) were observed in 66% of Utah (UT) counties, 60% of New Hampshire (NH) counties, and 31% of Colorado (CO) counties (Figure 3A). UT, NH, and CO are each in the top ten states with the highest number of recreation-dependent counties in the conterminous U.S., and UT and CO also contain many mining-dependent counties. Recreation-dependent counties had the highest median IWI and second highest HWBI (Figures S2B and S3B). This may indicate that people afforded the services that contribute to high HWBI may also have an increased opportunity to participate in outdoor recreational activities supported by intact watersheds in their own or nearby counties within their state. While we do not definitively conclude that watershed-based recreation contributes to increased well-being in these counties of UT, NH, and CO, studies have shown a relationship between the two (Abraham et al., 2010). Accordingly, our preliminary research suggests that it may be worthwhile to explore opportunities to catalyse improvements to well-being by investing in recreation in other parts of the U.S. Industrial and economic diversity may also be important in these states, with UT and CO having the highest (1.63) and sixth-highest (1.56) diversity of county industry-dependence among all states of the conterminous U.S., respectively, as determined using the Shannon Diversity Index (Figure S5). NH has a relatively low Shannon diversity of county industry-dependence (1.05); however, this is likely due to the small number of counties in this state. Other factors not examined in this study may also contribute to these ‘win-win’ situations; for example, good environmental governance and/or strong institutions.

Relatively intact watersheds but struggling communities (high mean IWI and low HWBI) were observed in 63% of counties in the desert and semi-arid ecoregions of northern Arizona (AZ), New Mexico (NM), and south–western Texas (TX) (Figure 3B). NM and AZ have the two highest proportions of government industry-dependence of all states in the conterminous U.S. (39 and 33% of counties, respectively), exceeded only by the District of Columbia. This coincides with the significant association between government industry-dependence and low HWBI in the spatial regression model. Counties dependent upon government industry also had increased mean IWI in the regression model, suggesting that government industry does not impair watershed integrity in the same way that industrial farming does, for example, so the potential provision of watershed ecosystem services in the deserts of AZ, NM, and TX should be intact. However, communities in this region have been supported by a long history of farming, largely through the use of traditional irrigation methods—*acequia*—which can maintain watershed integrity while ecosystem services (i.e., crop production) are utilised (Fernald et al., 2007). HWBI may, in fact, be underestimated in this region because the nationwide index is not particularly suited to capture the cultural, socio-economic, and demographic groups in this area (Smith et al., 2015). Further, the traditional ways in which they live are now under threat from increasing urbanisation and demand for water resources (Fernald et al., 2007), which also threaten watershed integrity; hence, these watersheds must be managed as social-ecological systems to maintain their biophysical integrity and increase local community well-being.

Thirty-seven percent of counties in eastern Kentucky (KY) and West Virginia (WV) also had relatively intact watersheds but struggling communities (Figure 3B). This region is heavily dependent upon mining and government industries. Over 65% of mining-dependent counties in eastern KY and WV had a HWBI below the 25^th^ percentile but mean IWI above the 75^th^. Although mountaintop mining is known to have cumulative detrimental impacts on stream ecosystems in this region (Lindberg et al., 2011), its effects on the physical integrity of watersheds (as defined in the IWI) may be confined to localised areas, and watershed integrity at larger scales appears to remain relatively high in this region (Figure 1A), despite potential water quality issues.

Communities with high well-being and heavily impaired watersheds (low mean IWI and high HWBI) were predominantly observed in the Temperate Prairies (44% of counties), particularly through Iowa (IA; 59%), Minnesota (MN; 39%), and North Dakota (ND; 34%) (Figure 3C). This ecoregion is dominated by farming-dependent counties, a dependence that can significantly reduce watershed integrity, based on the spatial regression results. Likely the environmental conditions in the Temperate Prairies that make them amenable to large-scale, intensive agriculture have been exploited to increase well-being at the detriment to watershed integrity. Whether such practices are sustainable in this region is of utmost importance. The Central USA Plains also had a high proportion of counties with low mean IWI and high HWBI (32%). Manufacturing and nonspecialised industry-dependence dominate this ecoregion, each representing 44% of counties, respectively. Although manufacturing industry-dependence did not significantly reduce mean county IWI overall, these counties had the second lowest median value behind farming-dependent counties (Figure S2B).

Finally, disadvantaged communities and degraded watersheds (low mean IWI and HWBI) dominated counties of the Mississippi Valley (Figure 3D). Sixty-three percent of counties in Arkansas (AR), Mississippi (MS), and Missouri (MO) that fell within the Mississippi Alluvial and Southeast USA Coastal Plains ecoregion had mean IWI and HWBI below the 25^th^ percentile. The Mississippi Valley part of this ecoregion has a clearly different social-ecological watershed condition than the coastal plains. No distinct associations with industry-dependence nor state could be discerned in this region, and these counties with low mean IWI and HWBI appear to be primarily associated with the physiographic region. A combination of historical and contemporary impacts on well-being and watershed integrity are likely to have caused the patterns observed in the Mississippi Valley; for example, a history of intensive cotton production, physical modifications to the river and floodplain, race, and socioeconomic status, which have similarly created high social vulnerability to natural hazards in this region (Cutter and Finch, 2008). This region is also an example of how local communities can be socially and environmentally disadvantaged to the benefit of individuals or communities elsewhere (Barry, 2007), which is discussed in the next section.

## Leveraging ecosystem services and the spatiotemporal characteristics of trade-offs

Spatial analysis and mapping of social-ecological water-shed conditions are first steps toward quantifying the interactions between ecosystem services and human well-being and beginning to manipulate these interactions and their trade-offs via adjustments in policy or management (Barnett et al., 2016; Nelson et al., 2009; Qiu and Turner, 2013). By deconstructing the HWBI into its 23 social, economic, and ecosystem service subindices within the groups of counties highlighted in Figure 3, we hypothesise that opportunities to leverage ecosystem services provided by intact watersheds in order to increase human well-being can be identified. We also hypothesise that the benefits provided by utilising watershed ecosystem services and the costs borne upon communities are spatially and temporally disconnected, with distant or downstream areas experiencing the impacts of exploitation upstream or historically. These spatial disconnections can be even more pronounced in the social realm (e.g., urban-rural) than in the biophysical realm (cf. Cronon, 2009). For employment, finance, activism, emergency preparedness, family services, healthcare, justice, and labor, the counties with high HWBI throughout UT, NH, CO, the Temperate Prairies, and the Central USA Plains had markedly higher scores than those counties with low HWBI in the desert southwest, eastern KY and WV, and the Mississippi Valley (Figure S6A and B). Ecosystem service subindex scores, however, were inconsistent with this pattern (Figure S6C). While these results suggest that the social and economic subindices drive the overall HWBI up or down in these statistically-outlying regions, particularly in terms of the social services provided, opportunities to improve the HWBI may exist in the ecosystem services realm (please see Summers et al., 2014 for detailed breakdown and maps of the HWBI sub-indices throughout the U.S.).

Specifically, opportunities to leverage watershed ecosystem services to increase well-being in eastern KY and WV may exist and are worthy of further investigation in the future. The HWBI water quantity subindex was generally high in the counties of eastern KY and WV that had low HWBI and high mean IWI (Figure S6C). High availability of water and high watershed integrity provide an excellent platform for recreation-based industry, which is common in UT, NH, and CO—areas with high HWBI and high mean IWI. The utilisation of ecosystem services provided by intact watersheds in eastern KY and WV may enable the development of recreation-based industry and the creation of jobs in the region. Increasing the diversity of industries within this region, and decreasing the reliance upon mining and government industries, may also improve well-being and bolster resilience against any potential collapse of the mining industry (Garmestani et al., 2006). Economic diversity is thought to increase the stability and adaptability of regional economies (Martin, 2012), reducing their susceptibility to collapse following an environmental disturbance (or social crisis, or both), which may be relevant for counties in eastern KY and WV. Although KY and WV have relatively high diversities of county industry-dependence at the state level (Figure S5), of the 33 counties in eastern KY and WV with low HWBI and high mean IWI, 17 (52%) are mining-dependent and 8 (24%) are government-dependent. These counties could better leverage their watershed-supported ecosystem services to draw outside economic value to the region, promoting jobs and a diversified economy beyond their mining- and government-dependence. However, good governance would be essential in such endeavours to ensure that resources are not only captured by elites or investors living in distant communities, but instead lead to improved well-being in local communities. Future investigation with the inclusion of the Gini coefficient, which is often used as an indicator of wealth or income inequality, would be valuable in order to account for social exploitation and inequality, for example by filling jobs with seasonal or migrant workers.

Historical exploitation of ecosystem services may have temporally lagged effects on human well-being in areas with heavily impaired watersheds, such as the Temperate Prairies and the Central USA Plains ecoregions. The HWBI air and water quality subindex scores were much lower in the Central USA Plains counties with high overall HWBI than in other regions (Figure S6C). Lower air and water quality would be expected in impaired watersheds because of a reduced ability to regulate atmospheric and aquatic emissions. Although the HWBI was high in many counties of the Central USA Plains, these areas may be at risk of future decline in well-being if provision of air and water quality ecosystem services are not restored in the heavily impaired watersheds of this region. Unexpectedly, the HWBI water quality and quantity subindex scores were not substantially lowered in the Temperate Prairies counties with high HWBI and low mean IWI (Figure S6C). In general, the Temperate Prairies were agriculturally developed after the Central USA Plains. These counties may experience a delayed decline in air and water quality ecosystem services, which would constitute a lagged effect of watershed exploitation (Liu et al., 2007). This study explored spatial patterns in watershed integrity and human well-being based on a single snapshot in time; however, the trajectories of these two indices over time is critically important. For example, if a threshold in the integrity of watersheds throughout the Temperate Prairies has been crossed, these watersheds may no longer be able to provide the ecosystem services upon which current agricultural practices have become dependent, and historical exploitation of resources may lead to future collapse of these industries. Landscape history is imperative in assessment of ecosystem service trade-offs (Tomscha and Gergel, 2016), which emphasises the importance of recurrent assessment of the social-ecological condition of watersheds over time (Parsons et al., 2016). Thus, these indices should be regularly updated and evaluated.

Commonly in watersheds, the costs of utilising ecosystem services are exported downstream and not borne by the same communities whom receive the benefits (e.g., Goolsby, 2000). The HWBI water quantity subindex was low in the counties of the desert southwest and the Mississippi Valley that had low HWBI (Figure S6C). Climatically, this would be expected in the desert southwest but not in the Mississippi Alluvial and Southeast Coastal Plains ecoregion—an area not typically characterised by a shortage of water. The Mississippi Valley is, however, characterised by a history of intensive water resource development both locally and in its upstream watershed, which has likely resulted in diminished provisioning of this ecosystem service. Upstream-downstream relationships are ubiquitous in watershed processes, and for watersheds to be considered ‘healthy’ the integrity of linked resources should not be degraded by upstream practices (Karr and Chu, 1998). However, these relationships are difficult to capture and quantify in social-ecological watershed assessments. A possible solution is to consider the costs and benefits of utilising ecosystem services in a spatiotemporal source-sink context to glean a more complete perspective of watershed-scale trade-offs (Bagstad et al., 2014; Scott Leibowitz, U.S. EPA, Corvallis, OR, pers. comm.). Cost-benefit analysis has been used to evaluate ecosystem services for human well-being, although with some criticisms (Wegner and Pascual, 2011). Source-sink analysis has been used for studies of the distribution and dynamics of nutrients, heavy metals, sediment, biomass, and genes in freshwater ecosystems (e.g., Burns and Kendall, 2002; Meade, 1982). Coalescing the two approaches, costs and benefits of utilising ecosystem services can be thought of as entities that interact between sources and sinks in space and time throughout watersheds. Penalties might be applied to source areas of costs to remediate the effects in sinks, and such a perspective may also have relevance for environmental justice concerns. The Mississippi River watershed would provide an excellent case study of such an application in the future, given the source-sink, cost-benefit spatial patterns between watershed integrity and human well-being evident in this region.

## Conclusions: Utilising available data to advance watershed science

Big data on the condition of watersheds and communities are becoming increasingly available in many countries, concomitant with novel paradigms and approaches to monitoring and assessment of watersheds as social-ecological systems. However, if the development of databases and social-ecological concepts continue independently, their full combined potential for advancing watershed science and management will remain unexploited. This study aimed to coalesce concepts of coupled, social-ecological systems with existing data in a watershed context. Aggregated metrics or indices, such as those used in this study, are useful in this context because they help capture fundamental aspects of system behaviour in a way that makes comparison and communication easy (USEPA, 2010). Yet, indices have their limitations. For example, collapsing multiple indicators into a single measure reduces transparency making it difficult to determine drivers useful for adjusting policy mechanisms and management decisions. Accordingly, analysts should provide additional information including underlying variables and/or subindices (e.g., Figure S6) to facilitate management decision making (Fiksel et al., 2012). Further, it is impossible to assume that these indices capture all pertinent information about a system; hence multiple indices are often necessary (USEPA, 2010).

The purpose for which indices were designed is also an important consideration in their use for examining watersheds as social-ecological systems. The HWBI used in this study was not specifically designed within a watershed context, and it could be refined to maximise relevance to watershed science. For example, indices of agricultural-and irrigation-dependence of local economies, along with other factors, have been used to evaluate the vulnerability of communities throughout the Murray-Darling Basin, Australia, to future climate, landscape, and political change (Stenekes et al., 2010). Similar data may be available from the U.S. economic census or other sources for inclusion into a HWBI refined specifically for watershed applications. The IWI is also currently limited due to the availability of data on some important watershed stressors throughout the conterminous U.S. For example, grazing extent and intensity have not been included in the current version of the index, although their effects on watersheds have been known for decades (Meehan and Platts, 1978). The omission of grazing stress in the IWI may have resulted in unrealistically high scores in the southwestern U.S. in this study. However, we wanted to utilise the data available and capitalise on the investments made in their creation. Social and cultural values are also important services provided by watersheds, and a social values assessment of watersheds of the U.S. would nicely complement the suite of existing and planned indices.

Finally, defining the mechanisms by which ecosystem services might be leveraged to increase human well-being and those by which trade-offs are transferred in space and time are important research directions (Birgé et al., 2016; Liu et al., 2016). We have identified four regions within the conterminous U.S. that are worthy of more detailed investigation. ‘Win-win’ situations (sensu Qiu and Turner, 2013), in which both IWI and HWBI are high, appear to be occurring in UT, CO, and NH, and policy and management lessons may be learnt from these states and applied elsewhere in order to increase watershed integrity and human well-being. Further investigation of the roles that governance and institutional support play in these ‘win-win’ areas is also important for learning lessons and making improvements elsewhere. Eastern KY and WV may be an excellent region to leverage ecosystem services in order to increase human well-being, particularly in the face of uncertainty in the mining industry, upon which the region's economy is currently dependent. However, good governance in such endeavours is essential so that the benefits of resource exploitation in these regions are not exported to already advantaged sectors of society, resulting in further inequality. The Mississippi River Basin presents an ideal study area to examine sources and sinks of the costs and benefits of utilising watershed ecosystem services. The Temperate Prairies and Central USA Plains ecoregions, in the upper part of the basin, and the lower Mississippi Valley are two regions that stand in glaring contrast in terms of their social-ecological watershed condition and the potential to utilise ecosystem services to increase well-being. Further export of the costs of ecosystem service exploitation from the upper basin to the lower Mississippi Valley may have dire consequences for the region and these spatiotemporal relationships should be carefully examined. We also identified relationships between watershed integrity and environmental and socio-economic drivers, which may prove useful in the development of large-scale watershed action plans or management strategies.

Other factors such as governance, institutional structures, and urban-rural, class, or racial dichotomies were not considered in this study, but are important and serve as challenges for future research. In addition, work must be done to help reconcile the different spatial boundaries of watersheds and social or administrative (e.g., county) systems. Cognisance of scale in defining and reconciling these boundaries is essential (Dollar et al., 2007; Post et al., 2007). Moreover, detailed qualitative and finer-scaled quantitative research is necessary to truly dissect the mechanisms that created the spatial patterns observed in this study, and to hypothesise how they may affect outcomes of future social and watershed decision making. While these remaining challenges form a framework for future research, this study provides a first step of many toward using available, spatially-explicit data to understand and manage watersheds as coupled, social-ecological systems.

## Data Accessibility Statement

All county-level data and R scripts used in this research are available on the U.S. Environmental Protection Agency's ScienceHub or directly from MWS.

## Supplementary Material

Supp Material
