# Supplementary material for: People and water: Exploring the social-ecological condition of watersheds of the United States
Source: Elementa (Wash D C). Author manuscript; Available in PMC 2018 Apr 19. (PMC5906808; doi:10.1525/elementa.189)
Supplement: Supp Material [file NIHMS954509-supplement-Supp_Material.pdf]

Supplemental material

People and water: Exploring the social-ecological condition of watersheds of the United States

Murray W. Scown[1,*], Joseph E. Flotemersch[2], Trisha L. Spanbauer[3], Tarsha Eason[4], Ahjond Garmestani[4], and Brian C. Chaffin[5]

[1]Formerly, ORISE Participant Research Program, U.S. EPA, Cincinnati, Ohio, US; currently, Lund University Centre for Sustainability Studies, Lund, Sweden

[2]National Exposure Research Laboratory, U.S. EPA, Cincinnati, Ohio, US

[3]NRC Research Associate, U.S. EPA, Cincinnati, Ohio, US

[4]National Risk Management Research Laboratory, U.S. EPA, Cincinnati, Ohio, US

[5]College of Forestry & Conservation, University of Montana, Missoula, Montana, US

*Corresponding author: murray.scown@lucsus.lu.se

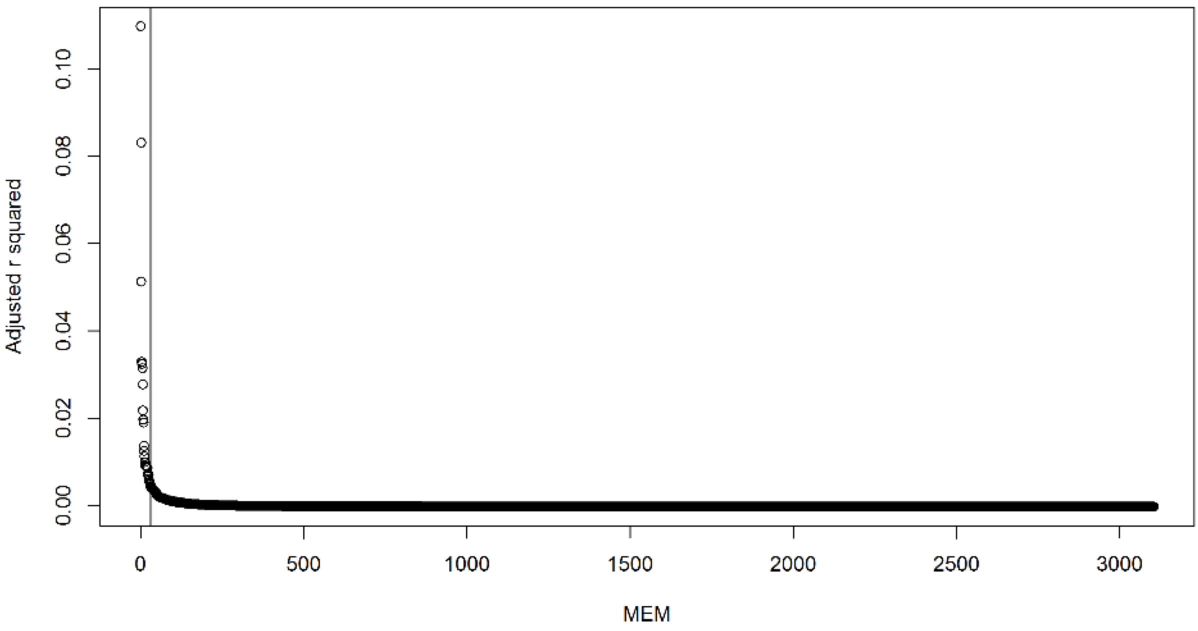

Figure S1A. Scree plot of variance in mean county IWI (adjusted r squared) explained by each Moran's Eigenvector Map (MEM). Vertical line indicates inflection point chosen as maximum number of MEMs to include in further analysis.

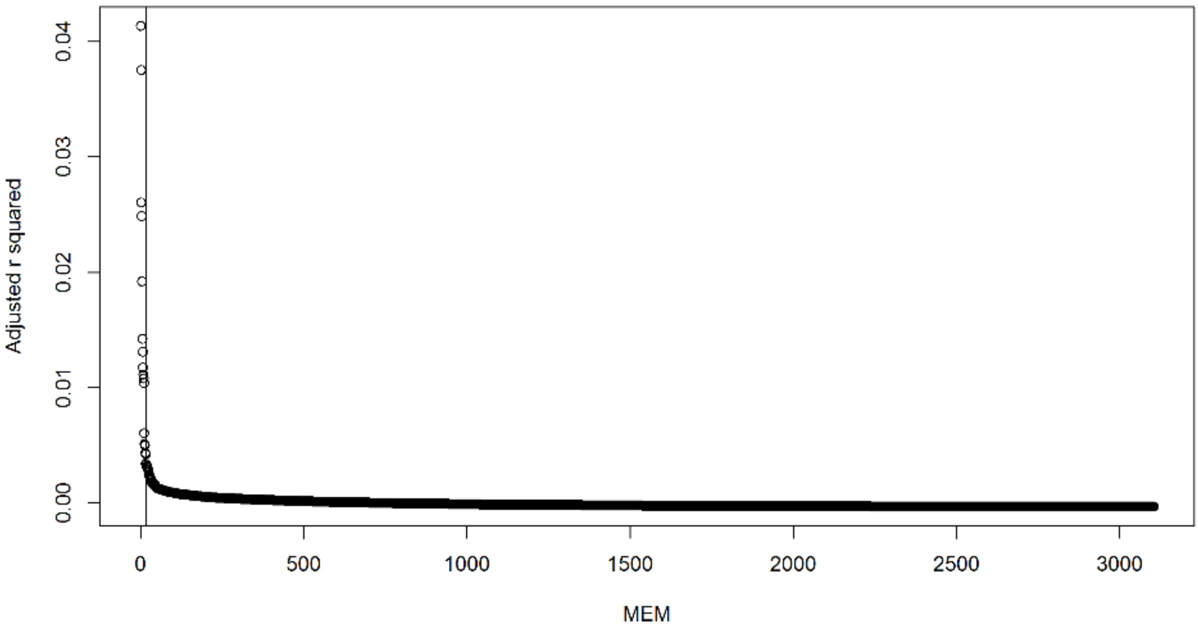

Figure S1B. Scree plot of variance in county HWBI (adjusted r squared) explained by each Moran's Eigenvector Map (MEM). Vertical line indicates inflection point chosen as maximum number of MEMs to include in further analysis.

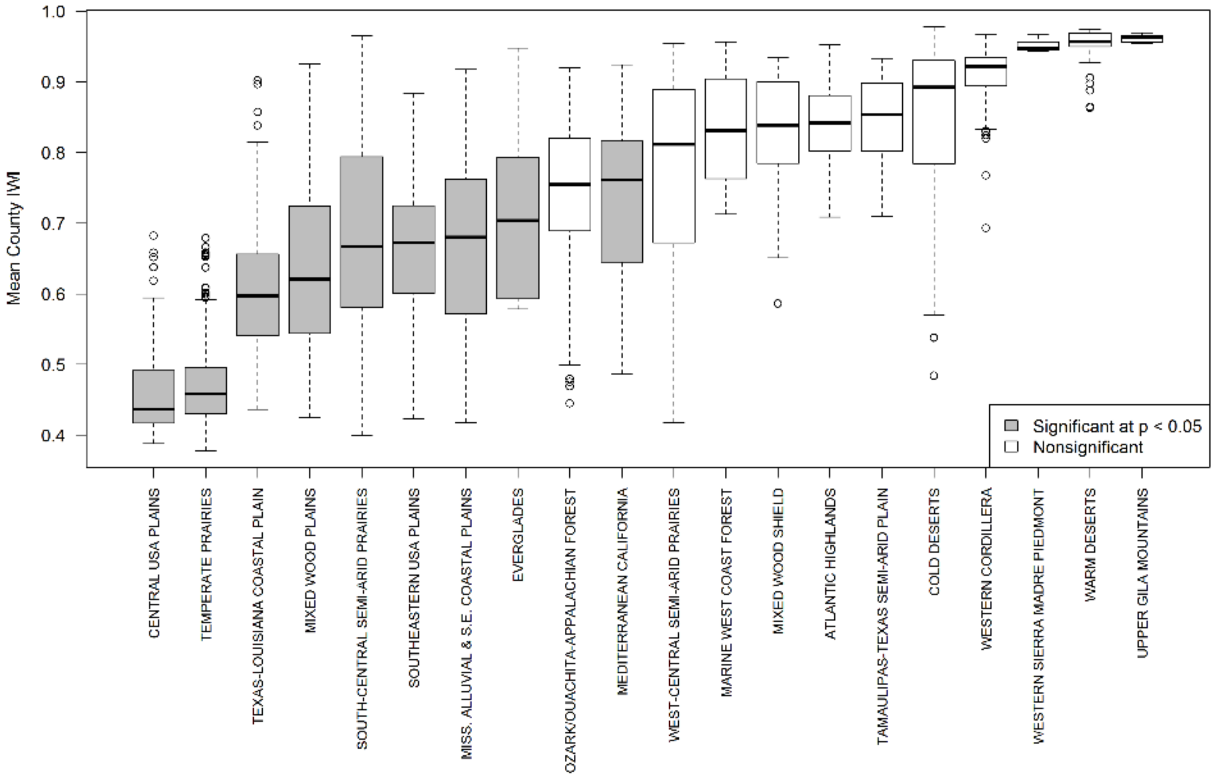

Figure S2A. Boxplot of mean county IWI scores within each level II ecoregion ordered by median. Shading indicates a significant effect in the spatial regression model. Dark line represents the median, box represents the first and third quartile, the whiskers represent +/- 1.58 times the quotient of interquartile range divided by $\sqrt{n}$, and the dots are values outside of this range. Also refer to Figure S2D for map of significant ecoregions.

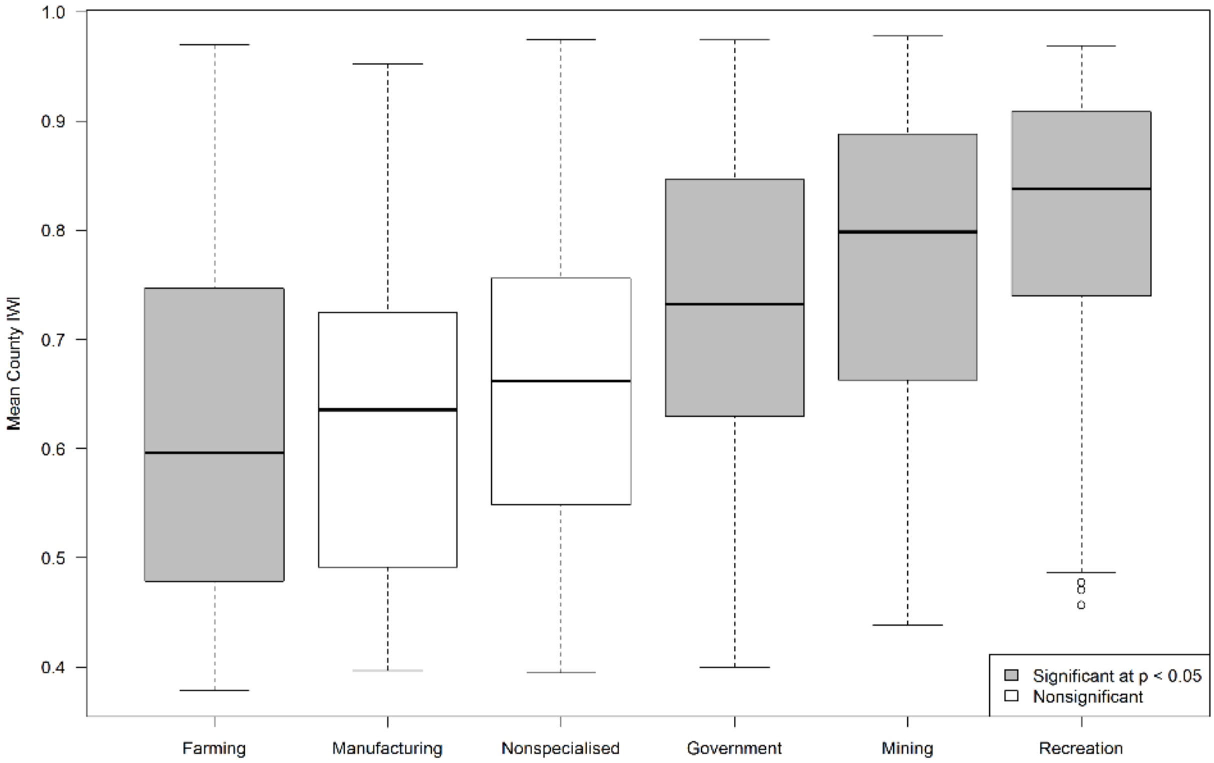

Figure S2B. Boxplot of mean county IWI scores within each industry-dependence category ordered by median. Shading indicates a significant effect in the spatial regression model. Dark line represents the median, box represents the first and third quartile, the whiskers represent +/- 1.58 times the quotient of interquartile range divided by $\sqrt{n}$, and the dots are values outside of this range. Also refer to Figure S2E for map of significant industry-dependence classes.

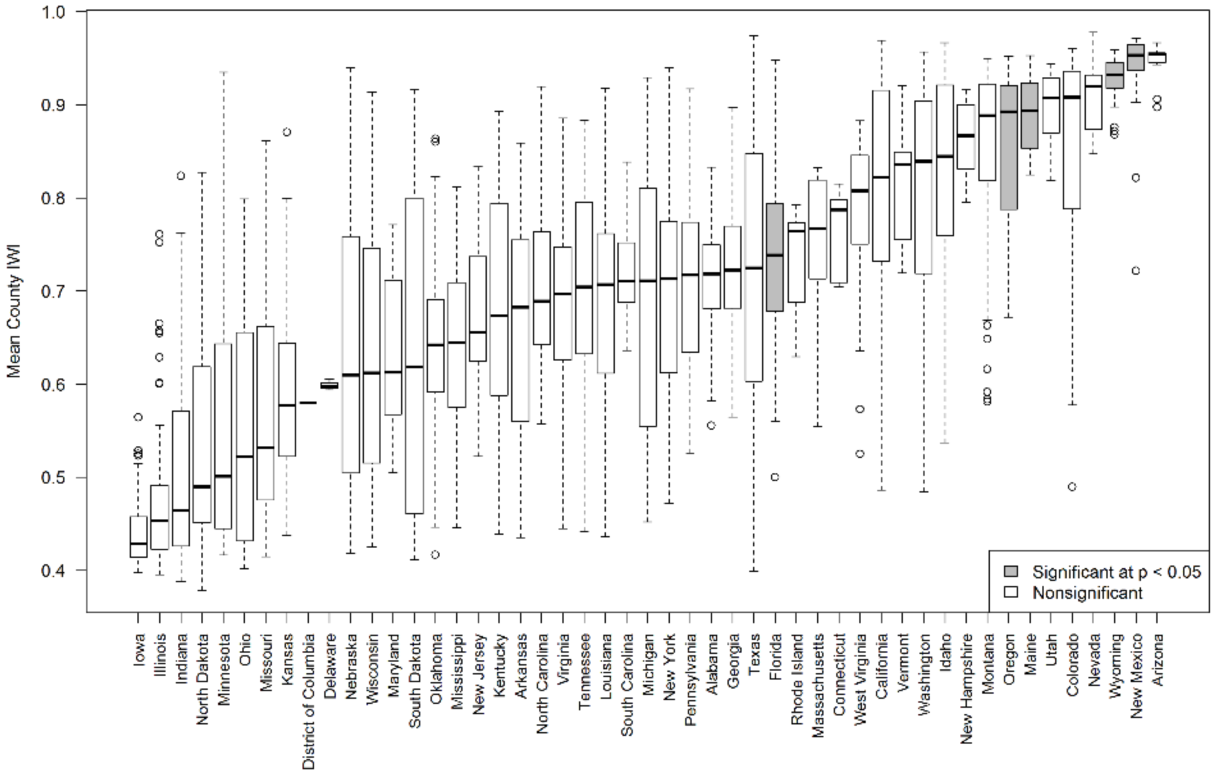

Figure S2C. Boxplot of mean county IWI scores within each state ordered by median. Shading indicates a significant effect in the spatial regression model. Dark line represents the median, box represents the first and third quartile, the whiskers represent +/- 1.58 times the quotient of interquartile range divided by $\sqrt{n}$, and the dots are values outside of this range.

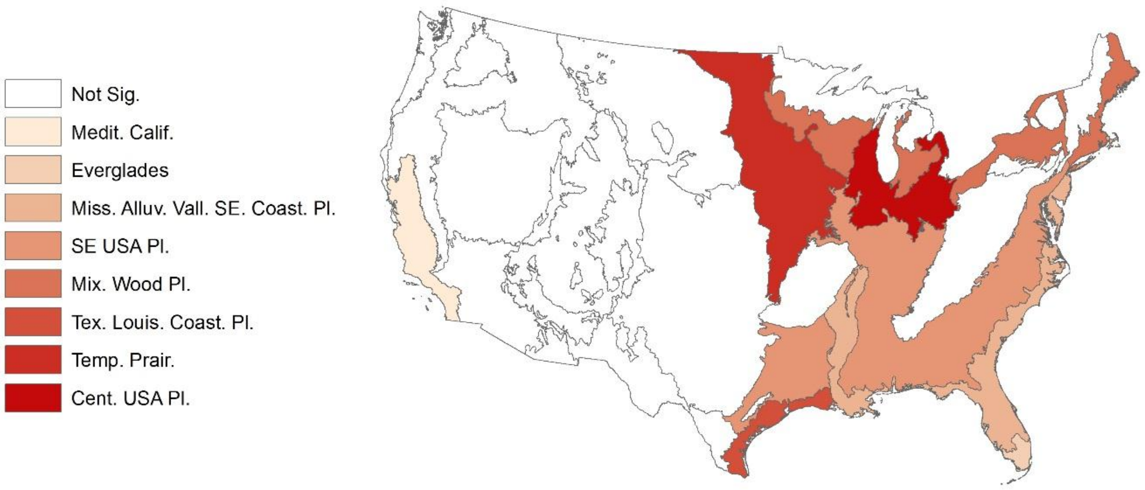

Figure S2D. Map highlighting the nine ecoregions that had a significant negative effect on mean IWI in the spatial regression models. Shading from dark to light based on lowest to highest median IWI among counties in these nine ecoregions.

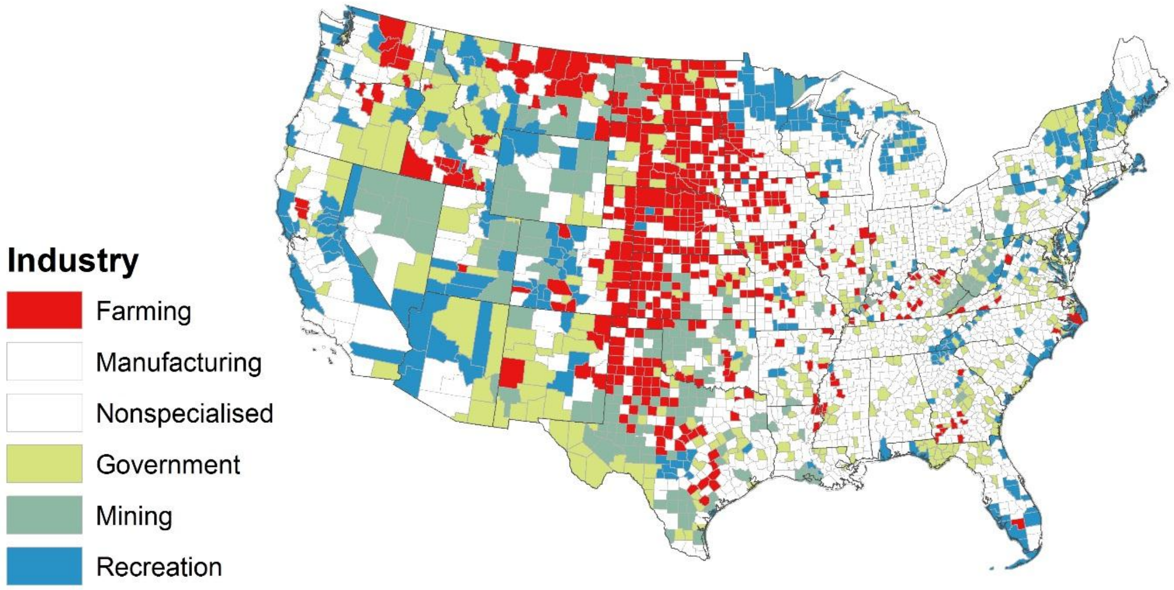

Figure S2E. Map highlighting the four industries that had a significant effect on mean IWI in the spatial regression models. Shading from red to blue based on lowest to highest median IWI among counties in these industry-dependence classes.

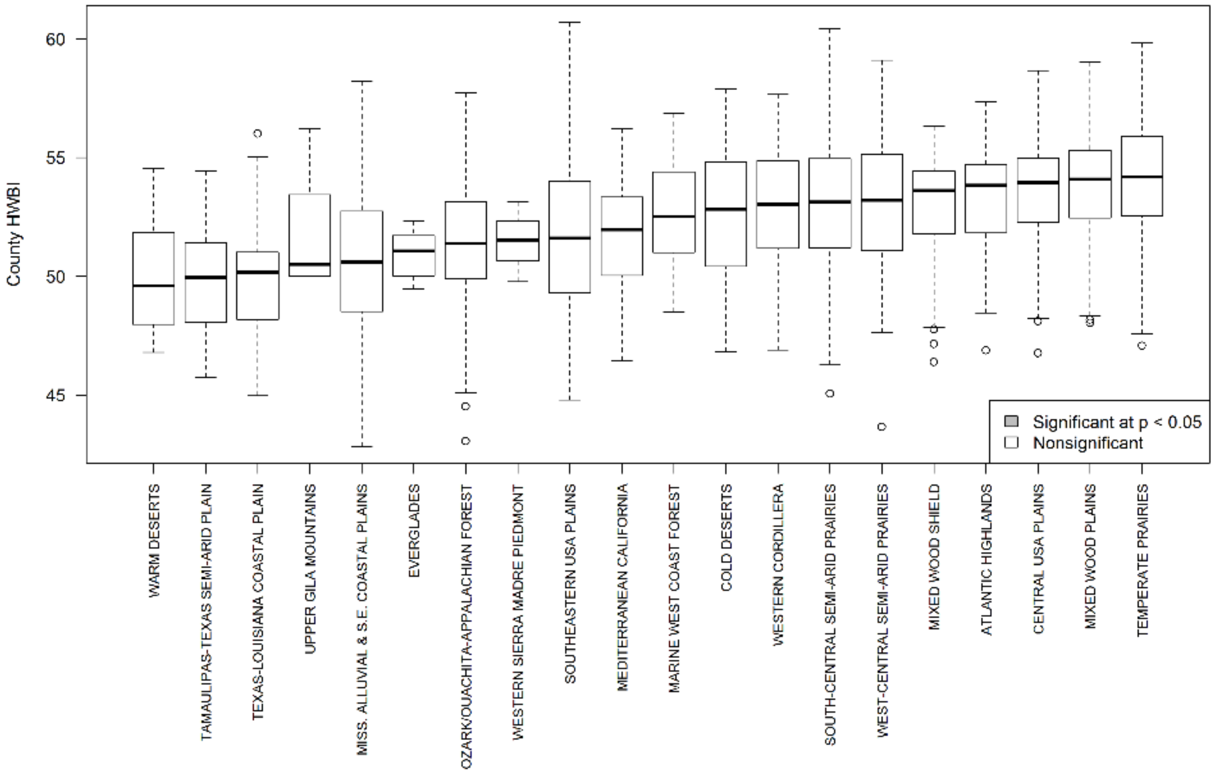

Figure S3A. Boxplot of county HWBI scores within each level II ecoregion ordered by median. Shading indicates a significant effect in the spatial regression model. Dark line represents the median, box represents the first and third quartile, the whiskers represent +/- 1.58 times the quotient of interquartile range divided by $\sqrt{n}$, and the dots are values outside of this range.

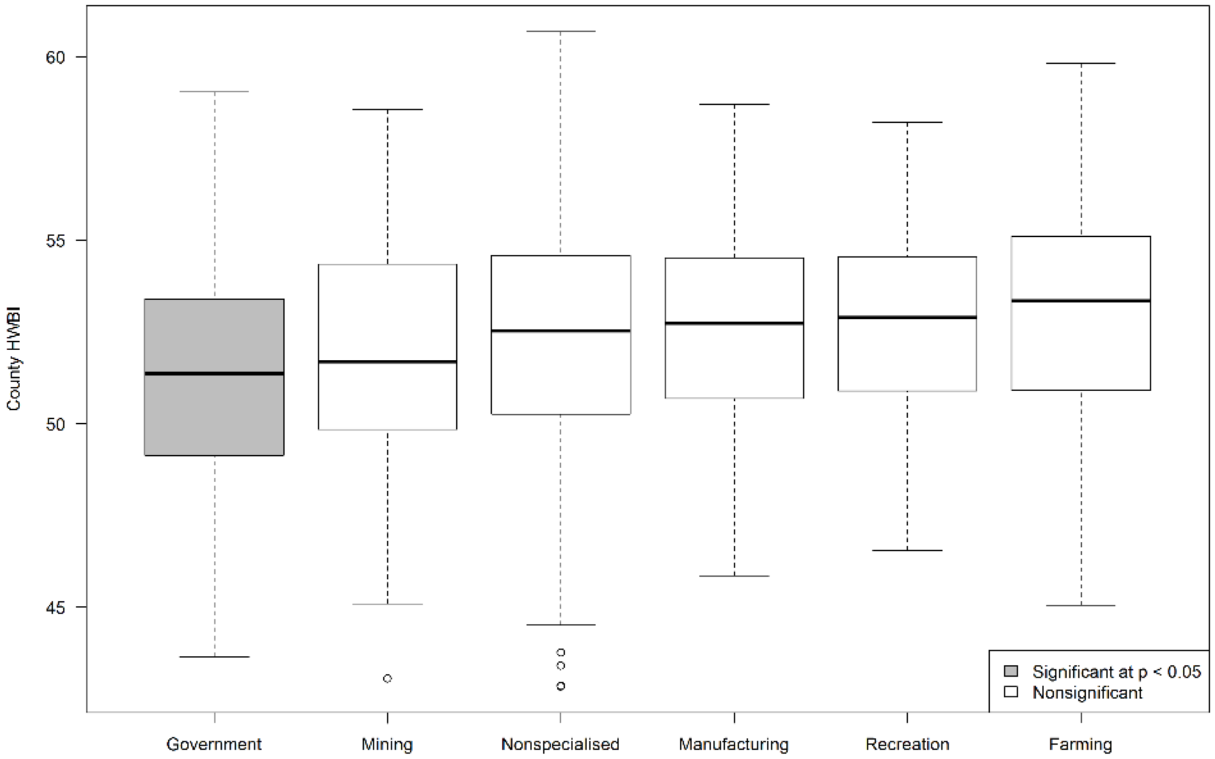

Figure S3B. Boxplot of county HWBI scores within each industry-dependence category ordered by median. Shading indicates a significant effect in the spatial regression model. Dark line represents the median, box represents the first and third quartile, the whiskers represent +/- 1.58 times the quotient of interquartile range divided by $\sqrt{n}$, and the dots are values outside of this range.

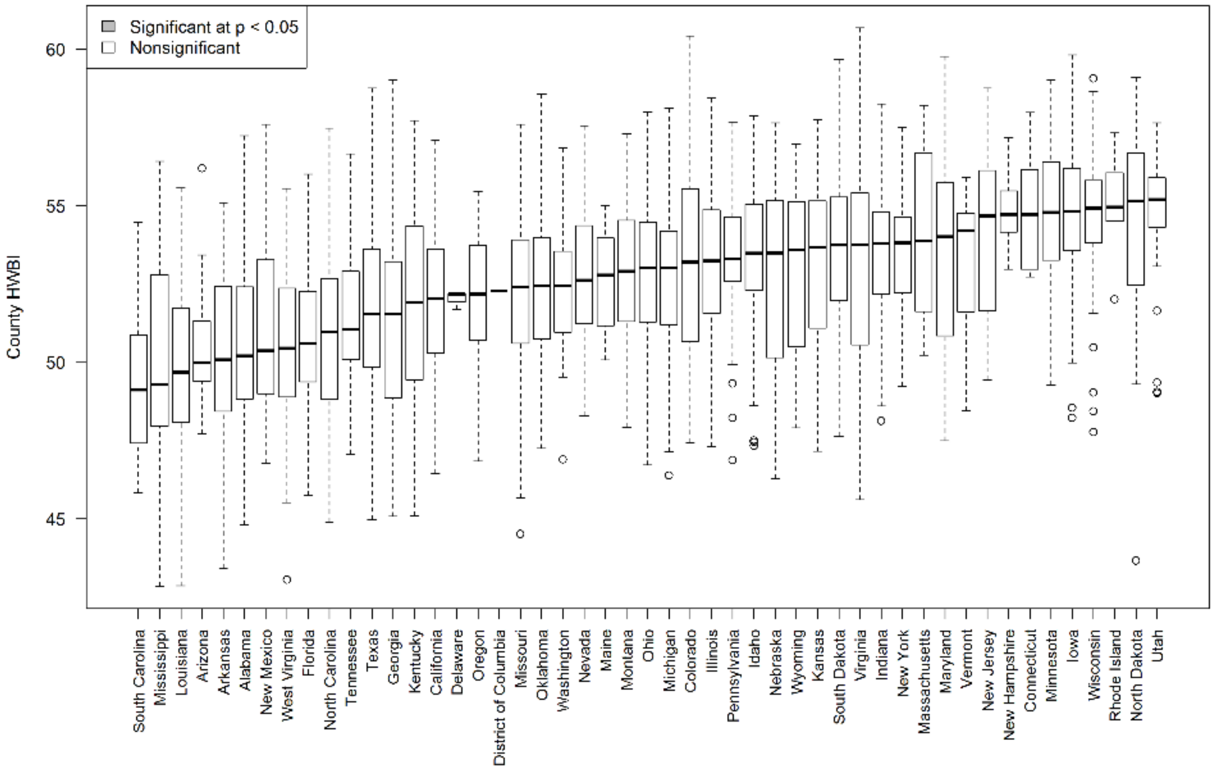

Figure S3C. Boxplot of county HWBI scores within each state ordered by median. Shading indicates a significant effect in the spatial regression model. Dark line represents the median, box represents the first and third quartile, the whiskers represent +/- 1.58 times the quotient of interquartile range divided by $\sqrt{n}$, and the dots are values outside of this range.

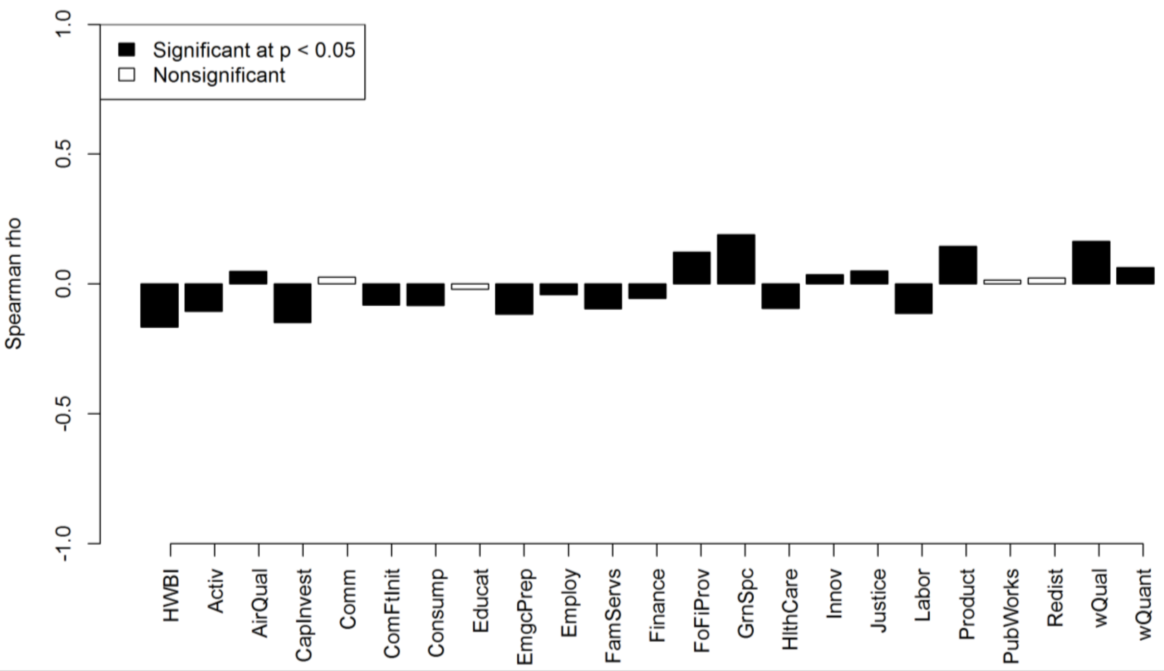

Figure S4. Spearman's rank correlation coefficient (rho) for mean county IWI against county HWBI and subindex scores for the conterminous U.S. Subindex names from left to right are activism, air quality, capital investment, communication, community and faith-based initiatives, consumption, education services, emergency preparedness, employment, family services, finance, food and fiber provisioning, greenspace, healthcare, innovation, justice, labor, production, public works, redistribution, water quality, and water quantity (see Summers et al., 2014 for details on HWBI subindices). Positive correlation coefficients indicate a positive relationship, negative coefficients indicate a negative relationship.

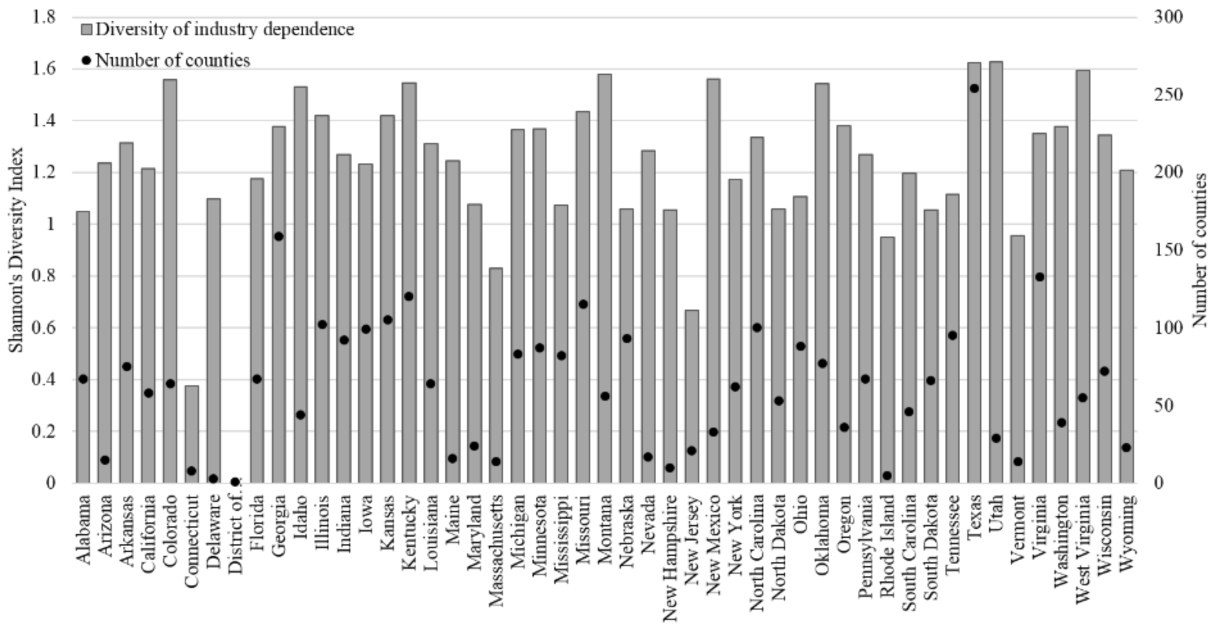

Figure S5. Shannon's Diversity Index of county industry-dependence within each state (left axis) and the number of counties within each state (right axis).

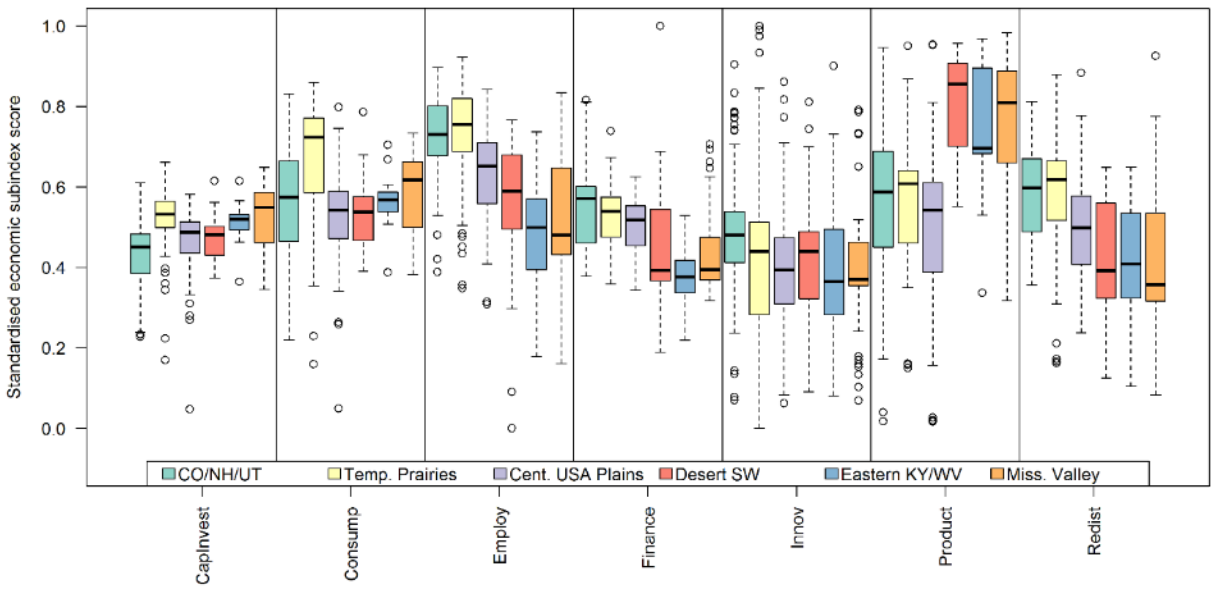

Figure S6A. HWBI economic service subindex scores for counties of particular regions highlighted in Figure 3 and discussed in the text. Subindex names from left to right are capital investment, consumption, education services, employment, finance, innovation, production, and redistribution (see Summers et al., 2014 for details on HWBI subindices).

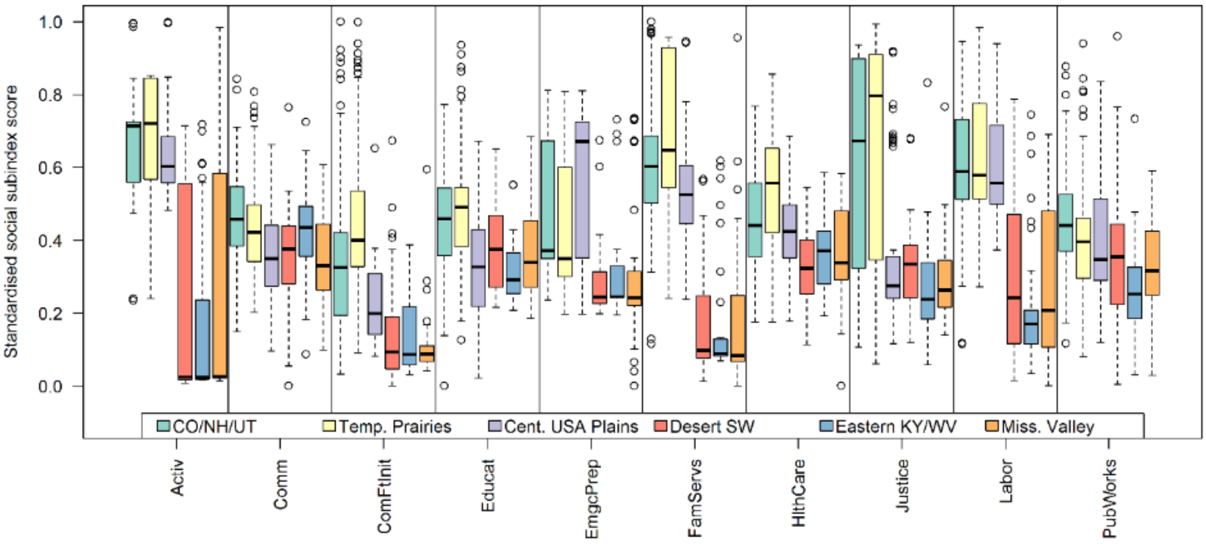

Figure S6B. HWBI social service subindex scores for counties of particular regions highlighted in Figure 3 and discussed in the text. Subindex names from left to right are activism, communication, community and faith-based initiatives, education services, emergency preparedness, family services, healthcare, justice, labor, and public works (see Summers et al., 2014 for details on HWBI subindices).

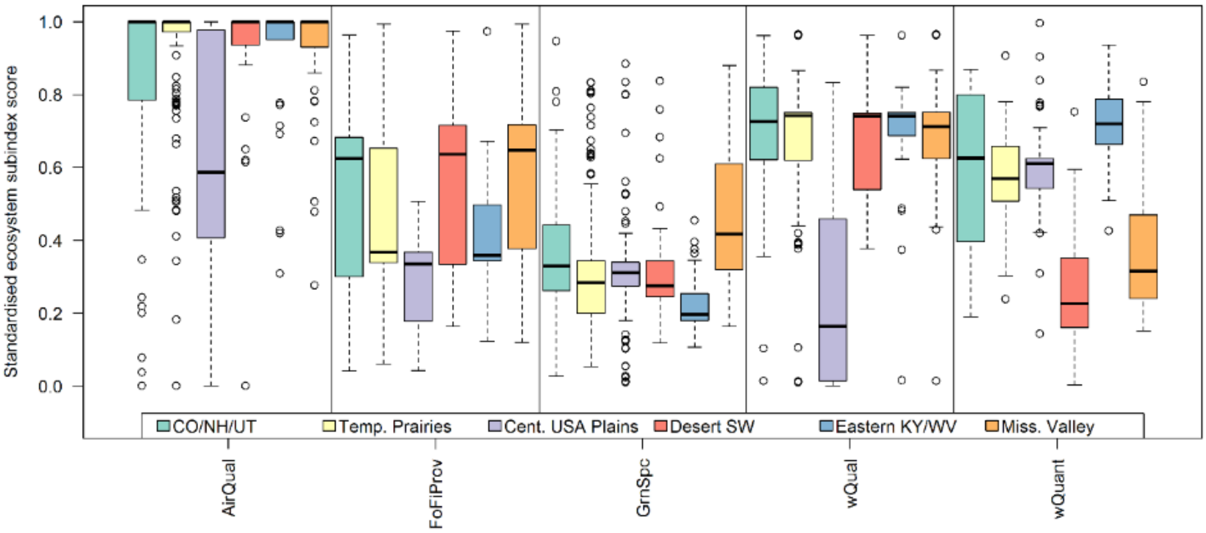

Figure S6C. HWBI ecosystem service subindex scores for counties of particular regions highlighted in Figure 3 and discussed in the text. Subindex names from left to right are air quality, food and fiber provisioning, greenspace, water quality, and water quantity (see Summers et al., 2014 for details on HWBI subindices).
